# Pathogenic role of lncRNA-MALAT1 in endothelial cell dysfunction in diabetes mellitus

**DOI:** 10.1038/cddis.2014.466

**Published:** 2014-10-30

**Authors:** J-Y Liu, J Yao, X-M Li, Y-C Song, X-Q Wang, Y-J Li, B Yan, Q Jiang

**Affiliations:** 1 Eye Hospital, Nanjing Medical University, Nanjing, China; 2 The Fourth School of Clinical Medicine, Nanjing Medical University, Nanjing, China; 3 Institute of Integrated Medicine, Nanjing Medical University, Nanjing, China

## Abstract

Long noncoding RNAs (lncRNAs) have important roles in diverse biological processes. Our previous study has revealed that lncRNA-MALAT1 deregulation is implicated in the pathogenesis of diabetes-related microvascular disease, diabetic retinopathy (DR). However, the role of MALAT1 in retinal vasculature remodeling still remains elusive. Here we show that MALAT1 expression is significantly upregulated in the retinas of STZ-induced diabetic rats and db/db mice. MALAT1 knockdown could obviously ameliorate DR *in vivo*, as shown by pericyte loss, capillary degeneration, microvascular leakage, and retinal inflammation. Moreover, MALAT1 knockdown could regulate retinal endothelial cell proliferation, migration, and tube formation *in vitro*. The crosstalk between MALAT1 and p38 MAPK signaling pathway is involved in the regulation of endothelial cell function. MALAT1 upregulation represents a critical pathogenic mechanism for diabetes-induced microvascular dysfunction. Inhibition of MALAT1 may serve as a potential target for anti-angiogenic therapy for diabetes-related microvascular complications.

Diabetes mellitus (DM) is a global health issue affecting children, adolescents, and adults. It could lead to organ and tissue damage in approximately one-third to one-half of people with diabetes.^1^ Diabetes is usually recognized as the vascular disease characterized by vasoregulation change, increased generation of reactive oxygen intermediates, inflammatory activation, and altered barrier function.^2^ Diabetic vascular complications, including microvascular and macrovascular dysfunction, occur in several organs, such as the muscle, eye, skin, heart, brain, and kidney.^3^ Regular screening and early detection of diabetic vascular complication is essential for reducing the morbidity and mortality of diabetic patients.

The retinal vasculature can be viewed directly and noninvasively, offering a unique and easily accessible window to study the health and disease of human vascular disease *in vivo*. In many diabetic patients, the primary retinal vascular complication – diabetic retinopathy (DR) – is well described.^4^ DR is characterized by progressive change in the retinal microvasculature, including increased retinal nonperfusion, enhanced vasopermeability, and pathological proliferation of retinal vessels.^3, 5^ Understanding the underlying mechanisms of retinal microvascular dysfunction has received considerable attention from the diabetes researchers and clinicians. Moreover, microvascular and macrovascular complications usually share several common characteristics. The easily accessible vessel of the eye may become a window to other types of diabetic-related vascular abnormality.

Long noncoding RNAs (LncRNAs) are recognized as the transcripts >200 nucleotides that structurally resemble mRNAs but do not encode proteins. LncRNAs participate in a variety of biological processes, such as chromosome imprinting, epigenetic regulation, cell-cycle control, cell apoptosis, and reprogramming of induced pluripotent stem cells.^6, 7^ Recently, Human *β* cell transcriptome analysis indicates that lncRNAs are dynamically regulated and abnormally expressed in type 2 diabetes.^8^ GWAS study shows that lncRNA-ANRIL is significantly associated with increased susceptibility to type 2 diabetes.^9^ These evidences indicate that lncRNA is a potential regulator of DM pathogenesis. However, the role of lncRNA in diabetic-induced vascular abnormality still remains elusive.

In our previous study, we established a mouse model of DR and performed lncRNAs' microarray to identify DR-related lncRNAs. The result suggests that lncRNA-MALAT1 (metastasis-associated lung adenocarcinoma transcript 1) is significantly upregulated in RF/6A cell model of hyperglycemia, in the aqueous humor samples, and in the fibrovascular membranes of diabetic patients.^10^ In this study, we investigated the role of lncRNA-MALAT1 in diabetic-induced retinal microvascular dysfunction.

## Result

### MALAT1 level is significantly upregulated in diabetic animal models

**Figure 1:**
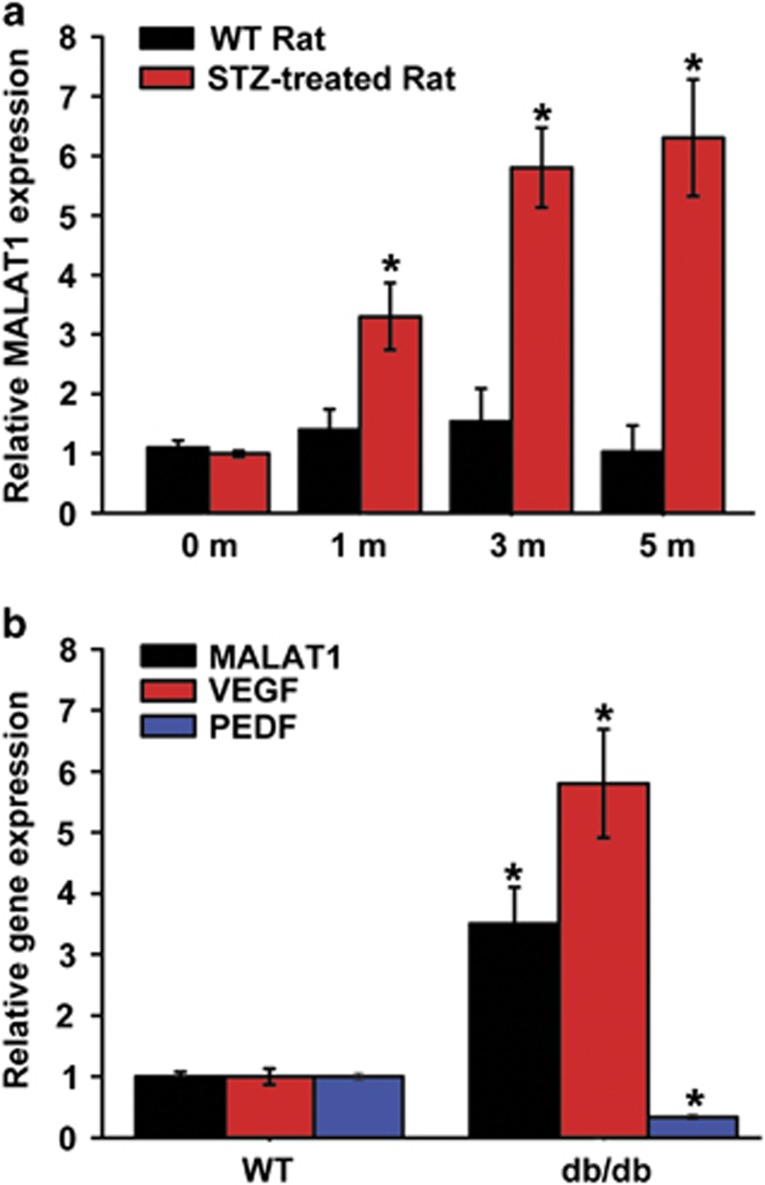
MALAT1 level is significantly upregulated in diabetic animal models. (**a**) Total RNAs were collected from the retinas of 1, 3, and 5 month after diabetes induction. Quantitative reverse transcriptase–PCRs (qRT-PCRs) were performed to detect MALAT1 levels in the retinas of STZ-induced diabetic rats and their respective non-diabetic controls. Relative MALAT1 expression was shown as the fold increase compared with the respective control (mean±S.E.M., *n*=6, *P<0.05). (**b**) qRT-PCRs were performed to detect the levels of MALAT1, vascular endothelial growth factor (VEGF), and PDEF in the retinas of db/db mice and their respective non-diabetic controls. Relative gene expression was shown as the fold increase compared with the respective control (mean±S.E.M., *n*=6, *P<0.05)

MALAT1 is a highly abundant and evolutionary conserved lncRNA. Our previous study shows that MALAT1 is significantly upregulated in the retinas of diabetic mice, in high-glucose-treated retinal endothelial cell line (RF/6A) or in the aqueous humor samples and fibrovascular membranes of diabetic patients.^10^ Here we further investigated the MALAT1 expression pattern in the diabetic animal models. Quantitative RT-PCR (qRT-PCR) shows that MALAT1 level is significantly higher in the retinas of streptozotocin (STZ)-induced diabetic rats, compared with that of non-diabetic rats (Figure 1a). MALAT1 level is also significantly upregulated in the retinas of db/db mice, a type 2 diabetic model, compared with their corresponding controls (Figure 1b). Together, these findings indicate that MALAT1 is a potential regulator of diabetes-induced microvascular complication.

### MALAT1 knockdown ameliorates retinal function in diabetic rats

Diabetes was induced in Sprague-Dawley rats by a single intravenous STZ injection. STZ injection results in hyperglycemia and a progressive loss of body weight. Compared with the diabetic wild-type rat, MALAT1 knockdown does not further affect body weight and blood glucose levels (Table 1).

**Figure 2:**
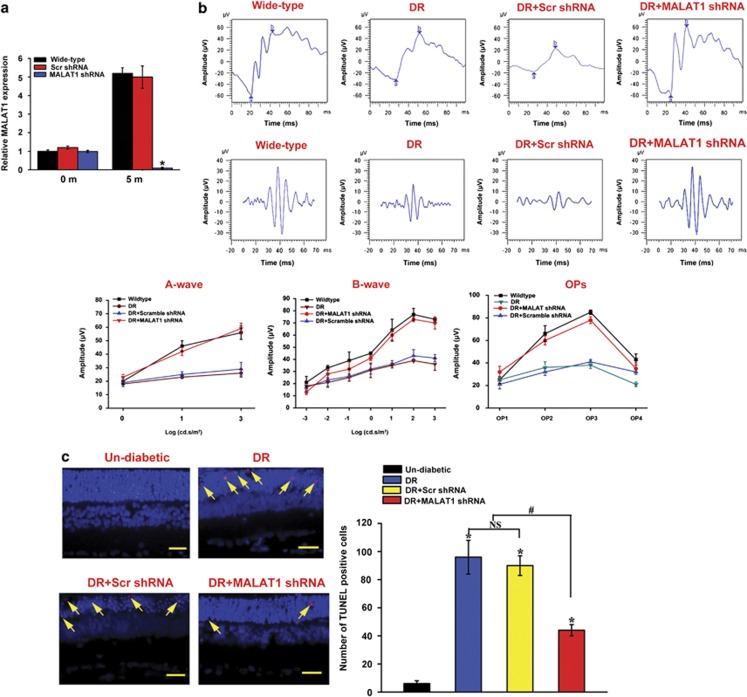
MALAT1 knockdown ameliorates retinal function in diabetic rats. (**a**) Rats received an intraocular injection of Ad-Scr shRNA or MALAT1 shRNA and then were injected with STZ for diabetes induction. MALAT1 levels in different experimental groups were detected using quantitative reverse transcriptase–PCRs. (**b**) Five months after diabetes induction, electroretinogram (ERG) was recorded in anesthetized rats, including non-diabetic rats (Wide-type rats), diabetic rats, and diabetic rats, that received an intraocular injection of Ad-Scr shRNA or MALAT1 shRNA. The representative ERG and OP-waves for different experimental groups are shown. Amplitudes of a-, b-, and OP-waves were statistically analyzed. (**c**) A representative image of TUNEL (terminal deoxinucleotidyl transferase-mediated dUTP-fluorescein nick end labeling) assay and quantitative result of TUNEL-positive cells are shown. TUNEL-positive retinal cells are indicated with yellow arrowhead. Blue color: DAPI stained nuclei. Red color: TUNEL-positive cells. The scale bars represent 50 *μ*m. *Indicates a significant difference compared with the control group. ^#^Indicates a significant difference between the marked experimental groups. The data shown are from a group size of *n*=6 animals

To reveal the effect of MALAT1 knockdown on visual function, visual electrophysiology was conducted in wild-type rats (un-diabetic rats), diabetic rats, and diabetic rats with an intraocular injection of scramble shRNA or MALAT1 shRNA adenovirus. qRT-PCR shows that MALAT1 shRNA but not scramble shRNA injection significantly downregulates MALAT1 level throughout the experiment (Figure 2a). The amplitudes of a-, b- and oscillatory potential (OP)-waves were found to be substantially reduced in the diabetic animals 5 months after diabetes induction. MALAT1 knockdown significantly ameliorates retinal function and reverses the decrease trend of a-, b- and OP-waves (Figure 2b), suggesting that MALAT1 knockdown prevents ERG abnormality induced by DM.

Apoptosis contributes to retinal cell loss upon hyperglycemia stress. TUNEL assay was performed to detect the number of apoptotic retinal cells 5 months after diabetes. Compared with the age matched non-diabetic controls, hyperglycemia results in a significant increase in the number of TUNEL-positive cells. Intraocular injection of the scramble shRNA does not further change the apoptosis percentage of retinal cells, whereas MALAT1 knockdown could obviously decrease the number of apoptotic retinal cells (Figure 2c).

### MALAT1 knockdown alleviates retinal vessel impairment in diabetic rats

**Figure 3:**
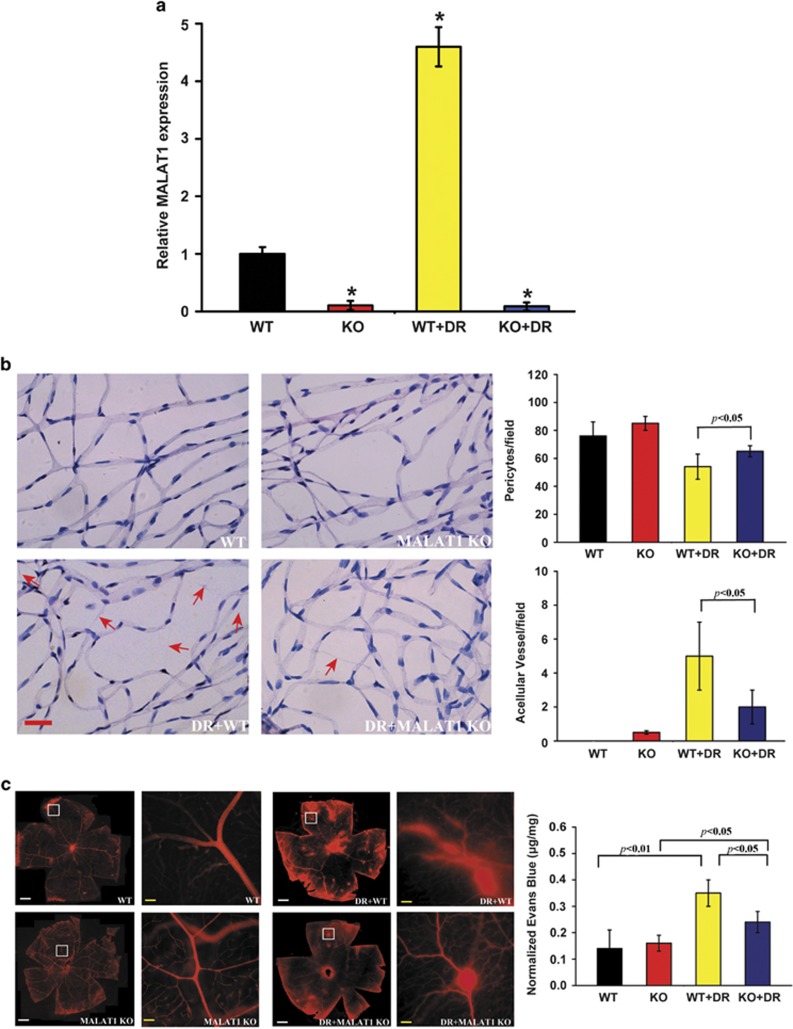
MALAT1 knockdown alleviates retinal vessel impairment in diabetic rats. (**a**) Diabetes was induced in MALAT1 KO rats and wild-type control by STZ injection. MALAT1 levels in different experimental groups were detected using quantitative reverse transcriptase–PCRs. *Indicates a significant difference compared with the un-diabetic wild-type group. (**b**) Five months after diabetes onset, retina trypsin digestion was performed to detect the number of pericytes and acellular capillaries. Red arrows indicate acellular capillaries. Pericytes and acellular capillaries were quantified in 10 random fields per retina and averaged (*n*=6). Scale bar: 25 *μ*m. (**c**) The rats were treated as shown in panel (**a**). These animals were infused with Evans blue dye for 2 h. The fluorescence signaling of flat-mounted retinas was detected using a fluorescence microscope. A representative image is shown. Quantification of Evans blue leakage was conducted in the experimental groups as shown. Data are shown as mean±S.E.M. for each group and representative of three independent experiments (*P<0.05, Student's *t*-test). White scar bar: 100 *μ*m; Yellow scar bar: 25 *μ*m

To examine the role of MALAT1 in retinal vessel dysfunction, retina trypsin digestion was performed to detect the change of retinal microvascular system, including pericytes and acellular capillaries. qRT-PCR shows that MALAT1 level is significantly reduced by MALAT1 injection but not scramble shRNA injection (Figure 3a). We found that there is no significant difference in the number of pericytes and acellular capillaries between non-diabetic wild-type and non-diabetic MALAT1 knockdown rats. Diabetes leads to a severe pericytes loss and aggravated capillary degeneration, whereas MALAT1 knockdown could reverse this trend (Figure 3b).

Vascular leakage is a key feature of early stage of DR. We used the Evans blue-albumin method to detect diabetes-induced retinal vascular leakage. The result shows that there is no difference for retinal vascular permeability between non-diabetic wild-type rats and MALAT1 knockdown rats. Hyperglycemia could significantly increase retinal vascular leakage, whereas intraocular injection of MALAT1 shRNA could alleviate vascular leakage in diabetic rats (Figure 3c).

### MALAT1 knockdown alleviates retinal inflammation in diabetic rats

**Figure 4:**
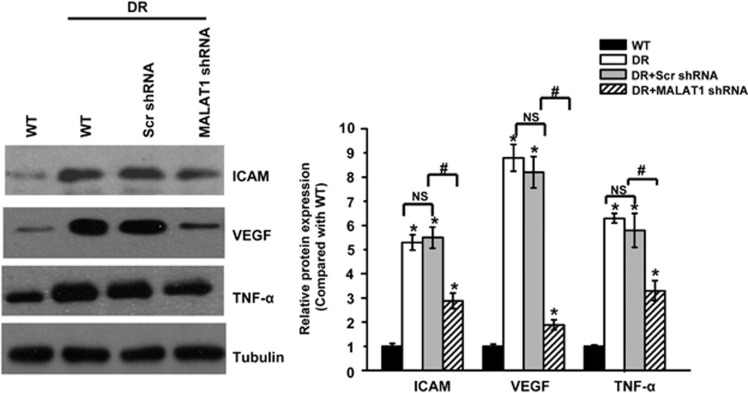
MALAT1 knockdown alleviates retinal inflammation in diabetic rats. Five months after diabetes onset, the same amount (30 *μ*g) of retina protein from different experimental groups was used for western blotting analysis of ICAM-1, vascular endothelial growth factor (VEGF), and TNF-*α*. Tubulin was detected as the loading control. A representative immunoblot was shown along with the quantitative data showing the mean± S.E.M. from four separate blots. *Indicates a significant difference compared with the un-diabetic wide-type group. ^#^Indicates a significant difference between the marked experimental groups. NS: no significant difference

Retinal inflammation has a key role in the development of diabetic microvascular complication. Pro-inflammatory proteins, such as intercellular adhesion molecule-1 (ICAM-1), VEGF, and tumor necrosis factor-*α* (TNF-*α*) have been reported to be upregulated during this pathologic process.^11^ We performed western blottings to detect the retinal expression of ICAM, VEGF, and TNF-*α*. Diabetes results in a marked increase in the expression of ICAM-1, VEGF, and TNF-*α*, whereas MALAT1 knockdown could significantly reduce the induction of VEGF, TNF-*α*, and ICAM-1 (Figure 4), suggesting that MALAT1 knockdown could alleviate retinal inflammation in diabetic rats.

### MALAT1 regulates the viability of retinal endothelial cells *in vitro*

**Figure 5:**
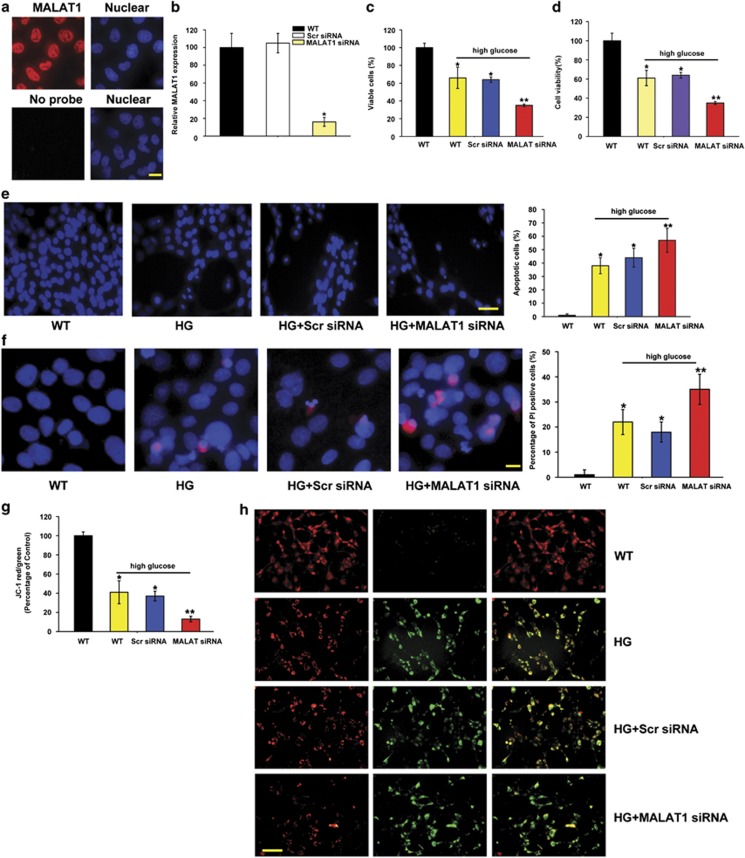
MALAT1 knockdown decreases the viability of retinal endothelial cell *in vitro*. (**a**) RNA fluorescence *in situ* hybridization was conducted to detect the distribution of MALAT1 in RF/6A cells. Scale bar: 10 *μ*m. (**b**) RF/6A cells were transfected with scramble siRNA (Scr), MALAT1 siRNA, or left untreated for 48 h. Quantitative PCR was conducted to detect MALAT1 level. (**c** and **d**) Viable cells were assessed by cell counting after trypan blue exclusion (**c**). Cell viability was detected using the MTT method (**d**). The data are expressed as the relative change compared with the wild-type group without high glucose treatment. (**e**) Apoptotic cells were analyzed using Hoechst staining and quantitated. The data are presented as means±S.E.M. and represented four individual experiments in which >500 cells were counted. Scale bar: 20 *μ*m. (**f**) Apoptotic cells were analyzed using PI staining and quantitated. Scale bar: 10 *μ*m. (**g** and **h**) RF/6A cells were incubated with JC-1 probe at 37 °C for 30 min, centrifuged, washed, transferred to a 96-well plate (100 000 cells per well), assayed using a fluorescence plate reader (**g**), and observed using a fluorescence microscope (**h**). Scale bar: 50 *μ*m. *P<0.05, **P<0.01, as analyzed by the Student's *t*-test

The pathological changes in diabetic microvascular complications are usually characterized by abnormal functions of endothelial cells.^3^ RNA fluorescence *in situ* hybridization (FISH) shows the enrichment of MALAT1 in the nuclear of RF/6A cells (Figure 5a). To gain the insight into the functional relevance of diabetes-induced MALAT1 upregulation, we estimated the effect of MALAT1 knockdown on endothelial cell viability *in vitro*. We found that MALAT1 small interfering RNA (siRNA) transfection leads to a significant reduction in MALAT1 level (Figure 5b). High glucose significantly decreases the number of viable cells (Figure 5c) and reduces the cell viability (Figure 5d) as detected by Trypan blue staining and 3-(4,5-dimethylthiazol-2-yl)-2,5-diphenyl tetrazolium bromide assay (MTT) assay. MALAT1 knockdown could further reduce cell viability of RF/6A cells (Figures 5c and d). To determine whether MALAT1 regulates the development of high-glucose-induced apoptosis, RF/6A cells were treated with MALAT1 siRNA, scramble siRNA, or left untreated, followed by high glucose treatment. Hoechst 33342, PI, and JC-1 staining was used to detect the apoptosis degree of RF/6A cells. The combination of MALAT1 knockdown and high glucose results in higher apoptotic percentage than high glucose alone, as shown by increased apoptotic nuclei (condensed or fragmented), more PI-positive cells (dying or dead cells), and decreased mitochondrial depolarization (Figures 5e–g). Immunofluorescence staining reveals that MALAT1 knockdown could accelerate the shift of fluorescence emission from green to red compared with these cells tranfected with the scramble siRNA upon high glucose stress (Figure 5h).

Excess glucose stress usually leads to abnormal oxidative stress in diabetic complications. Here we also treated RF/6A cells with H_2_O_2_ to mimic oxidative stress. We found that H_2_O_2_ treatment could significantly reduce cell viability, decrease the number of viable cells, and accelerate the development of apoptosis, whereas MALAT1 knockdown could further decrease the viability of RF/6A cells (Supplementary Figure S1). Taken together, these results suggest that MALAT1 has a critical role in the regulation of endothelial cell function *in vitro.*

### MALAT1 knockdown affects endothelial cell migration and tube formation *in vitro*

**Figure 6:**
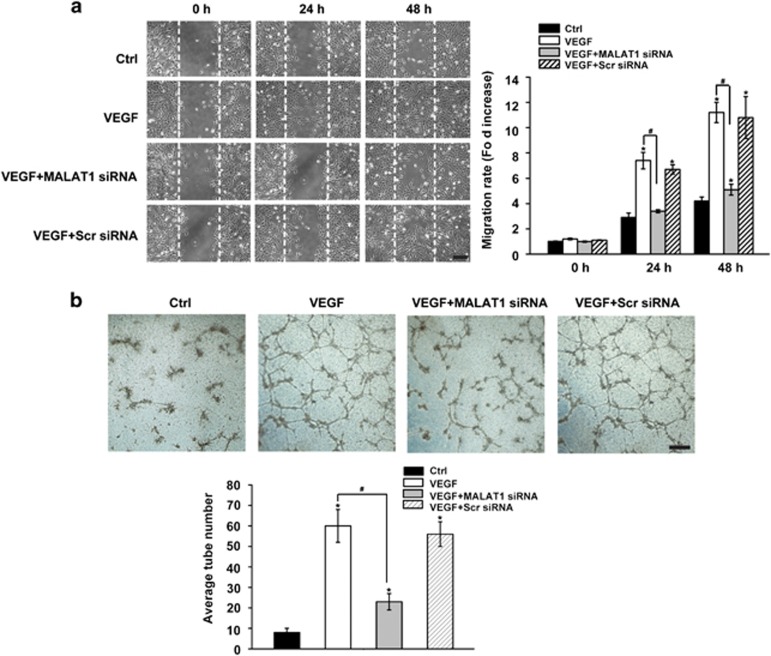
MALAT1 knockdown decreases retinal endothelial cell migration and tube formation *in vitro*. (**a**) RF/6A cells were transfected with MALAT1 siRNA, scramble siRNA (Scr), or left untreated and then stimulated with vascular endothelial growth factor (VEGF; 10 ng/ml). Cell migration was assessed using a wound-healing assay. Images of wounded monolayer were taken at times 0, 24, and 48 h after treatment with VEGF. The horizontal lines indicate the wound edge. Migration was estimated by measurement of cell numbers within the wounded region. The data are shown as the relative change compared the control group without VEGF treatment. A representative imageis shown. Scale bar: 100 *μ*m. (**b**) RF/6A cells were transfected with MALAT1 siRNA, scramble siRNA (Scr), or left untreated. These cells were seeded on the matrigel matrix and stimulated with VEGF. The tube-like structures was observed using a light microscopy 24 h after VEGF treatment in a blinded manner. The average number of tube formation for each field was statistically analyzed (*n*=50). *Indicates a significant difference compared with the corresponding control group. ^#^Indicates a significant difference between the marked experimental groups. A representative image is shown. Scale bar: 50 *μ*m

The upregulation of pathogenic factors, such as VEGF and TNF-*α*, has been reported during diabetes-induced microvasular dysfunction. Their increases have a critical role in endothelial cell migration and tube formation, thereby causing retinal vessel impairment.^11^ Here we investigated the effect of MALAT1 knockdown on VEGF- and TNF-*α*-induced endothelial cell migration and tube formation. We found that VEGF or TNF-*α* treatment could accelerate the migration of RF/6A cells. The transfection of scramble siRNA has no effect on VEGF or TNF-*α*-mediated cell migration. By contrast, MALAT1 knockdown could significantly reduce the number of migrated cells (Figure 6a and Supplementary Figure S2A).

A matrigel-based capillary-genesis assay was performed to assess the ability of RF/6A cells to form an organized tubular network. In the normal culture medium, minor cell organization was observed. VEGF or TNF-*α* treatment leads to a significant increase in tube formation, whereas MALAT1 knockdown significantly decreases the number of tube formation. By contrast, scramble siRNA transfection has no effect on VEGF- or TNF-*α*-induced endothelial capillary tube formation (Figure 6b and Supplementary Figure S2B).

### MALAT1 knockdown prevents hyper-proliferation of retinal endothelial cells through p38 mitogen-activated protein kinase (MAPK) signaling

**Figure 7:**
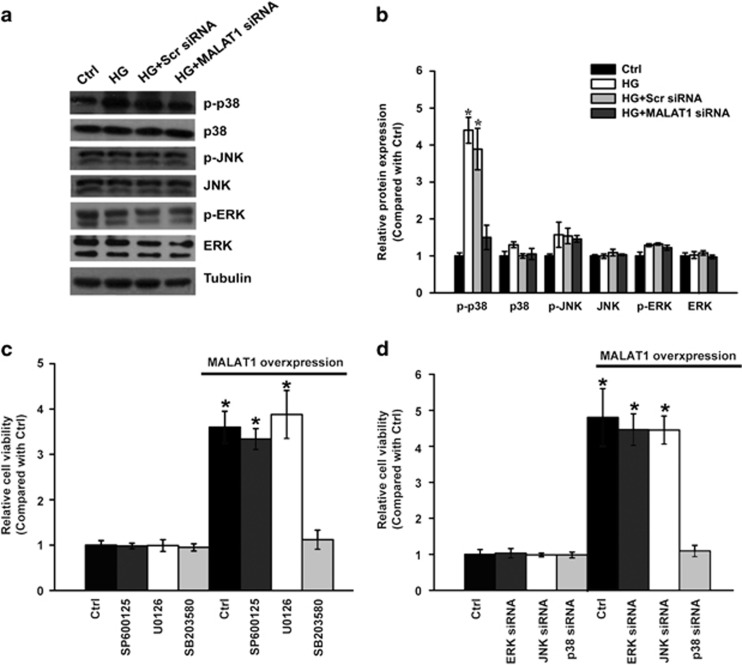
MALAT1 knockdown prevents the hyper-proliferation of retinal endothelial cells through p38 MAPK signaling. (**a** and **b**) RF/6A cells were transfected with MALAT1 siRNA, scramble siRNA (Scr), or left untreated and then exposed to high glucose for 48 h. The untreated group was taken as the control group. Representative immunoblots of total p38, total ERK, total JNK, p-p38, p-ERK, p-JNK, and tubulin (**a**) are shown along with the densitometric quantitative results (**b**). (**c**) RF/6A cells were transfected with MALAT1 to promote cell viability and treated with SB203580, U0126, or SP600125 for 48 h. Cell viability was detected using the MTT method (*n*=4). (**d**) RF/6A cells were co-transfected with MALAT1 and treated with p38 siRNA, ERK siRNA, or JNK siRNA for 48 h. The cell viability was detected using the MTT assay (*n*=4). All data are shown as mean±S.E.M. *Indicates a significant difference compared with the corresponding control group

In our previous study, bioinformatics analysis revealed that MAPK signaling is primarily involved in the pathogenesis of retinal neovascular disorders.^12^ We speculated that there is a potential crosstalk between MALAT1 and MAPK signaling during diabetes-induced retinal vessel impairment. Here we conducted western blotting to examine the activation status of MAPK signaling *in vitro*. We found that MALAT1 knockdown causes an obvious reduction of phosphorylated p38 level, but has no effect on the levels of phosphorylated ERK1/2 or JNK1/2 (Figures 7a and b), suggesting that MALAT1 knockdown leads to the inaction of p38 MAPK signaling. To examine whether p38 is involved in MALAT1-induced cell hyper-proliferation, MALAT1 was transfected into RF/6A cells to accelerate the proliferation of retinal endothelial cells. We found that pretreatment of RF/6A cells with SB203580, a p38 MAPK pathway inhibitor, strongly blocks the effect of MALAT1-induced RF/6A cell proliferation, whereas ERK inhibitor U0126 or the JNK inhibitor SP600125 has no effect on MALAT1-mediated change in cell viability (Figure 7c). Moreover, similar results were obtained when p38 MAPK activity was inhibited by p38 siRNA but not by ERK siRNA or JNK siRNA (Figure 7d). Taken together, these results suggest that MALAT1 regulates the hyper-proliferation of retinal endothelial cells through p38 MAPK signaling.

## Discussion

The mammalian genome harbors thousands of lncRNA genes. They have gained widespread attention in recent years due to the potential roles in many biological processes and disorders. Recently, the role of lncRNAs in vascular biology has been gradually recognized. RNA sequencing of human coronary artery smooth muscle cells identified 31 un-annotated lncRNAs. Of them, lncRNA-SENCR is found to be a vascular cell-enriched lncRNA, and its knockdown results in decreased expression of Myocardin and smooth muscle contractile genes.^13^ The knockdown of Ang II-regulated lncRNA, lnc-Ang362, affects the proliferation of vascular smooth muscle cells, suggesting a potential role in the diagnosis of Ang II-associated cardiovascular diseases.^14^ MALAT1 knockdown tips the balance from a proliferative to a migratory endothelial cell phenotype *in vitro*, and its genetic deletion or pharmacological inhibition could reduce vascular growth *in vivo*.^15^ CVD-associated lncRNA, ANRIL, have a critical role in atherosclerosis progression by modulating the expression of genes involved in cell proliferation, apoptosis, extra-cellular matrix remodeling, and inflammatory response.^16^ Our previous study reveals a potential link between MALAT1 and DR.^10^ In this study, we found that MALAT1 could activate p38/MAPK signaling pathway and regulate retinal endothelial cell function and pathological microvascular growth under diabetic condition. Taken together, the emergence of lncRNAs as regulators of gene expression would undoubtedly alter our understanding of the complex regulation network of pathological angiogenesis.

DR is one of the most common vascular complications in patients with long-term diabetes. Visual deterioration is tightly related with retinal inflammation, retinal neovascularization, vascular hyperpermeability, and the apoptosis of vascular cells.^17, 18^ Hyperglycemia is the most important characteristic of diabetes and exerts adverse effects on vascular cells during the progression of diabetic vascular complications. We found that hyperglycemia causes a significant upregulation of lnc-MALAT1 level in retinal endothelial cell and diabetic retinas. Silencing of MALAT1 significantly alleviates diabetic-induced retinal vascularization, vascular leakage, and retinal inflammation. Furthermore, genetic ablation of MALAT1 *in vitro* inhibits the proliferation, migration, and tube-formation ability of endothelial cells, typical aspects of endothelial cell biology. Overall, our findings indicate that MALAT1 is able to rescue the retina from hyperglycemia-induced degeneration and significantly improve retinal visual function.

MALAT1 was originally identified as an lncRNA, showing high expression in individuals at high risk for metastasis of non-small cell lung tumor. Its expression is significantly upregulated in a wide range of tumors, such as lung cancer, liver cancer, renal cell carcinoma, bladder cancer, and osteosarcoma.^19^ Previous studies have revealed that MALAT1 has a positive role in tumor cell proliferation, apoptosis, migration, invasion, or the metastatic spread of tumor cells.^20^ Here, we found that MALAT1 knockdown results in a significant reduction in retinal endothelial cell proliferation, migration, and tube formation. These data show that the mechanisms by which MALAT1 controls cell proliferation may be similar between tumor cells and endothelial cells.

MAPK signaling pathway is usually activated by many extracellular stimuli, including high glucose stress. MAPK activation has a series of physiological effects, such as apoptosis, cell proliferation, mitosis, and several gene transcription.^21, 22^ Aberrant MAPK activity could lead to continuous cellular proliferation or muted responses to external stimuli. Because of its versatile role in biological processes, MAPK signaling pathway has been implicated in many human disorders ranging from cancer to obesity.^22^ Here, we found that MALAT1 knockdown could significantly change the levels of phosphorylated p38 MAPKs but has no effect on the levels of phosphorylated ERK1/2 or JNK1/2. MALAT1-induced cell proliferation could be specifically blocked by p38 MAPK pathway inhibitor or p38 siRNA. All evidences indicate that there is a crosstalk between MALAT1 and p38 MAPK signaling. MALAT1-regualted MAPK signaling would affect a wide variety of cellular processes of retinal endothelial cells.

In conclusion, this study reveals the involvement of lncRNA-MALAT1 in diabetes-induced microvascular dysfunction. MALAT1 is significantly upregulated in diabetic animal models. Its knockdown significantly alleviates diabetic-induced microvasular dysfunction *in vivo* and inhibits endothelial cell proliferation, migration, and tube formation *in vitro*. Diabetes-induced MALAT1 upregulation represents an important pathogenic mechanism for diabetic microvascular complication. Inhibition of MALAT1 may serve as a novel therapeutic strategy for diabetes-related microvascular complications.

## Materials and Methods

### Ethics statement

All experiments were conducted in accordance with the ARVO Statement for the Use of Animals in Ophthalmic and Vision Research. All experimental procedures were approved by the Animal Care and Use Committee of Nanjing Medical University, Nanjing, China.

### Induction of diabetes in rats

Rats were fasted for 24 h before diabetes induction. Diabetes was induced by an intraperitoneal injection of STZ (60 mg/kg STZ in 10 mM citrate buffer, pH 4.5). Animals that served as non-diabetic controls received an equivalent amount of citrate buffer alone. Forty-eight hours later, rats with blood glucose levels >16.7 mmol/l were considered diabetic.

### RNA FISH

To detect MALAT1 expression, RF/6A cells were fixed in 4% formaldehyde for 15 min at room temperature and then permeabilized with 0.5% Triton X-100 on ice for 10 min. Cells were washed in PBS 3 × for 10 min and rinsed once in 2 × SSC prior to hybridization. Hybridization was carried out using Cy3-labeled cDNA probe in a moist chamber at 37 °C for 8 h.

### TUNEL analysis

Enucleated eyes were fixed in 4% paraformaldehyde for 3 h. After dehydration in a graded ethanol series, these samples were embedded in paraffin. Cryosections were cut in the sagittal plane through the optic nerve head. The sections were stained using fluorescein-conjugated TUNEL *in situ* cell death detection kit (Roche Diagnostics, Mannheim, Germany). The fluorescence image was obtained using the Olympus IX-73 fluorescence microscope (Olympus, Tokyo, Japan).

### Measurement of retinal vascular permeability

Blood–retinal barrier breakdown was detected using the albumin leakage from retinal blood vessels.^23^ Briefly, under deep anesthesia, Evans blue was injected through the tail vein at a dose of 40 mg/kg. After the dye circulated for 2 h, the chest cavity was opened, and the left heart ventricle was cannulated. Each rat was perfused with citrate-buffered 1% paraformaldehyde for 5 min to clear the dye. After the perfusion, the retinas were dissected and dried for weighing. Evans blue dye was extracted from the tissue by incubating each sample in 120 *μ*l formamide (Sigma, St. Louis, MO, USA) for 18 h at 70 °C. The extract was centrifuged at 10 000 × *g* for 30 min at 4 °C. The absorbance of the supernatant was measured at 620 nm. The concentration of Evans blue was calculated from a standard curve and normalized to the dry weight of retina.

### Retinal trypsin digestion

Trypsin digestion was performed to analyze retinal vasculature.^5, 18^ The eyes were fixed in 10% neutral-buffered formalin for 24 h. Retinas were incubated with 3% trypsin until the medium became cloudy and the tissues showed signs of disintegration. The retinas were shaken gently to free the vessel network from adherent retinal tissue, washed in fresh water, and mounted on glass slides to dry. These retinas were stained with PAS/hematoxylin. Endothelial cell nuclei are large and ellipsoid, whereas pericytes nuclei are smaller and darker situated on the outer side of vessel wall. A digital imaging system (FAS-1000; Toyobo, Osaka, Japan) was used to analyze four quadrants per retina for histopathological changes of retinal vasculature.

### Cell viability assay

The viability of RF/6A cells was assessed using MTT. Briefly, RF/6A cells were plated at a density of 1 × 10^4^ cells per well in 96-well plates. After specific treatment, these cells were incubated with MTT at a final concentration of 0.5 mg/ml for 3 h at 37 °C. After medium removal, 100 mM DMSO solution was added to dissolve the formazan crystals. The absorbance at 570 nm wavelength was detected using a microplate reader (Molecular Devices, Sunnyvale, CA, USA).

### Measurement of mitochondrial membrane potential (Δ*ψ*m)

RF/6A cells were incubated with the fluorescent cationic dye, JC-1(10 *μ*g ml^−1^; Molecular Probes), at 37 °C for 30 min to detect the mitochondrial membrane potential using a fluorescence plate reader (Molecular Devices) with excitation at 485 nm and collection of emission spectra between 530 and 620 nm. In the control cells, an intact Δ*ψ*m allowed JC-1 bearing a delocalized positive charge showing red fluoresces. In the apoptotic cells, the collapse of Δ*ψ*m caused JC-1 to remain in the cytoplasm in a green fluorescent monomeric form. Mitochondrial depolarization was detected by a decrease in the red-to-green fluorescence intensity ratio.

### RNA interference

Phosphorothioate internucleosidic linkage-modified antisense oligonucleotides (with 5 2'-O-methoxyethyl nucleotides on the 5′ and 3′ ends and 10 consecutive oligodeoxy nucleotides to support RNase H activity) were used to silence the MALAT1 expression. The oligonucleotides were transfected to cells two times (48 h) within a gap of 24 h, at a final concentration of 50 nM, using lipofectamine RNAi max reagent according to the manufacturer's instructions (Invitrogen, Carlsbad, CA, USA). Knockdown of p38, JNK, or ERK was conducted using double-stranded siRNAs (Dharmacon, Lafayette, CO, USA). The siRNA sequences will be provided upon request.

### Tube formation assay

Approximately 200 *μ*l basement membrane matrix (BD Biosciences, San Jose, CA, USA) was placed into each well of a 24-well plate. They were hardened for 30 min at 37 °C. RF/6A cells (2 × 10^4^) were plated on the top of BMM-coated wells. They were incubated with and without VEGF or TNF-*α* for tube stabilization for 24 h at 37 °C and observed using an Olympus IX-73 microscope.

### Statistical analysis

Data are expressed as mean±S.E.M. Statistical significance was analyzed by the Student's *t*-test or one-way ANOVA using SPSS 13.0 (SPSS Inc., Chicago, IL, USA). A probability value *P*<0.05 was considered statistically significant.

## Figures and Tables

**Table 1:** General physiological parameters of diabetic and non-diabetic rats

	**Initial**		**2 months**		**5 months**	
	**Body Wt (g)**	**Glucose (mg/dl)**	**Body Wt (g)**	**Glucose (mg/dl)**	**Body Wt (g)**	**Glucose (mg/dl)**
Un-diabetic	124±15	65±5.3	215±16	72±8.2	408±26	82±4.8
Diabetic	118±6.5	265±12*	175±8*	287±10.2*	315±29*	251±14.6*
Diabetic+Scr shRNA	132±7.8	278±10.5*	167±12.1*	258±21*	305±22.7*	265±7.9*
Diabetic+MALAT1 shRNA	121±8.5	262±9.5*	197±9.6	266±13.6*	308±16.9*	260±10.3*

Data are shown as mean±S.E.M. *Indicates a significant difference compared with the age-matched un-diabetic group

## Abbreviations

DM: diabetes mellitus
DR: diabetic retinopathy
lncRNA: long noncoding RNA
MALAT1: metastasis-associated lung adenocarcinoma transcript 1
siRNA: small interfering RNA
FISH: fluorescence *in situ* hybridization
MAPK: mitogen-activated protein kinase
OP: oscillatory potential
ICAM-1: intercellular adhesion molecule-1
TNF-*α*: tumor necrosis factor-*α*
STZ: streptozotocin
