# Supplementary figures and images for: Pathogenic role of lncRNA-MALAT1 in endothelial cell dysfunction in diabetes mellitus
Source: Cell Death Dis. 2014 Oct 30;5(10):e1506–. doi: 10.1038/cddis.2014.466 (PMC4649539; doi:10.1038/cddis.2014.466)

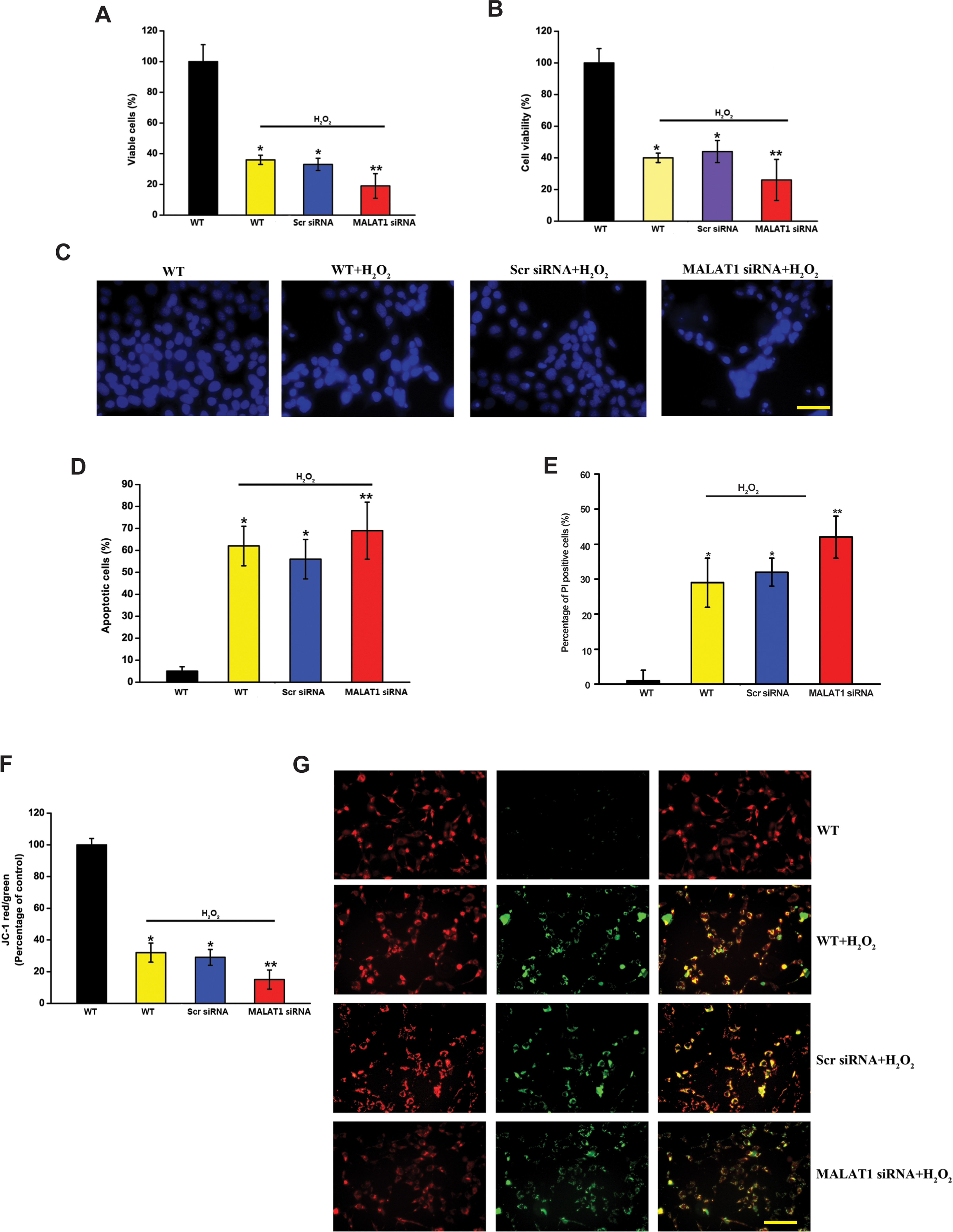

Supplement: Supplementary Figure S1 [file cddis2014466x1.tif]

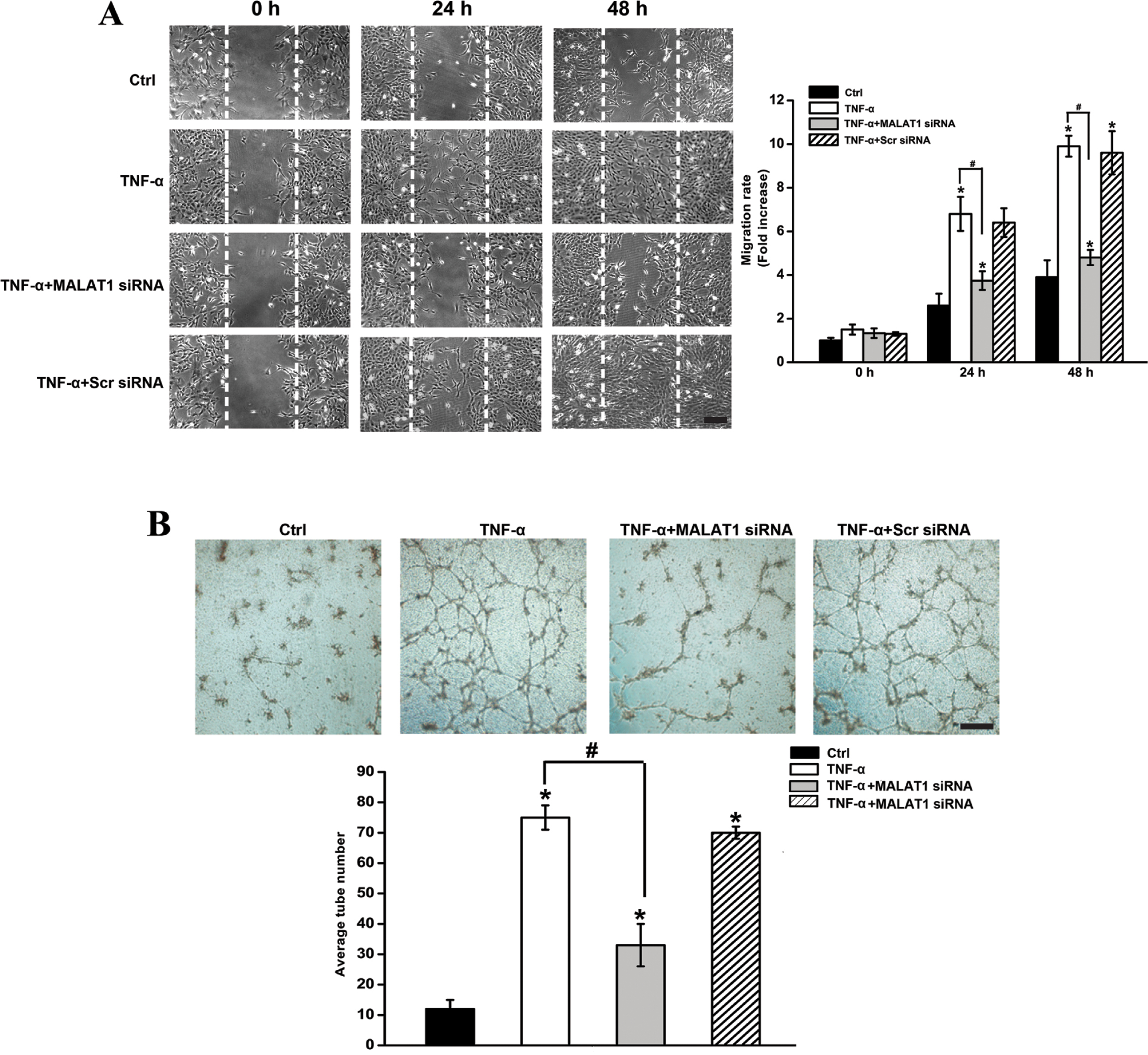

Supplement: Supplementary Figure S2 [file cddis2014466x2.tif]
