# Supplementary material for: Pathogenic role of lncRNA-MALAT1 in endothelial cell dysfunction in diabetes mellitus
Source: Cell Death Dis. 2014 Oct 30;5(10):e1506–. doi: 10.1038/cddis.2014.466 (PMC4649539; doi:10.1038/cddis.2014.466)
Supplement: Supplementary Figure Legends [file cddis2014466x3.doc]

**Fig S1: MALAT1 regulates the viability of retinal endothelial cell upon oxidative stress *in vitro***

(A) RF/6A cells were treated as shown. Viable cells were assessed by cell counting after trypan blue exclusion. The data was expressed as the relative change compared with wild-type group without H_2_O_2_ treatment. (B) Cell viability was detected using MTT method. The data was expressed as the relative change compared with wild-type group without H_2_O_2_ treatment. (C and D) RF/6A cell apoptosis was induced using H_2_O_2_ incubation. Apoptotic cell was analyzed using Hoechst staining (C) and quantitated (D). The results were presented as means ±S.E.M., and represented four individual experiments in which more than 500 cells were counted. Scale bar: 20 μm. (E) Apoptotic cells were analyzed using PI staining and quantitated. (F and G) RF/6A cells were treated as shown, and apoptosis was induced by H_2_O_2_ treatment. These cells were incubated with JC-1 probe at 37 °C for 30 min, centrifuged, washed, transferred to a 96-well plate (100,000 cells per well) and assayed using a fluorescence plate reader (F) and observed using a fluorescence microscope (G). Scale bar: 50 μm. **P*<0.05, ***P*< 0.01, as analyzed by Student's *t* test.

**Fig S2: MALAT1 knockdown affects TNF-α-induced endothelial cell migration and tube formation *in vitro***

(A) RF/6A cells were transfected with MALAT1 siRNA, scramble siRNA (Scr), or left untreated, and then stimulated with TNF-α (10 ng/ml). Cell migration was assessed using a wound-healing assay. Images of wounded monolayer were taken 0, 24, and 48 h after treatment with TNF-α. The horizontal lines indicate the wound edge. Migration was estimated by measuring cell numbers within the wounded region. The data was shown as the relative change compared the control group without TNF-α treatment. A representative image was shown. Scale bar: 100 μm. (B) RF/6A cells were transfected with MALAT1 siRNA, scramble siRNA (Scr), or left untreated. These cells were seeded on the matrigel matrix, and stimulated with TNF-α. The tube-like structures was observed by light microscopy 24 h after TNF-α treatment in a blind fashion. The average number of tube formation for each field was statistically analyzed (n = 50). “^*^” indicates a significant difference compared with the corresponding control group. “^#^” indicates a significant difference between the marked experimental groups. A representative image was shown. Scale bar: 50 μm.
